# Real-time ultrasound-guided external intracerebral hemorrhage drain placement

**DOI:** 10.1186/s40779-020-00261-9

**Published:** 2020-07-02

**Authors:** Cong Feng, Sai Huang, Li Chen, Xuan Zhou, Li-Li Wang, Fa-Qin Lv, Tan-Shi Li

**Affiliations:** 1 Department of Emergency, the First Medical Center of Chinese PLA General Hospital, Beijing, 100853 China; 2 Department of Hematology, the First Medical Center of Chinese PLA General Hospital, Beijing, 100853 China; 3 Department of Ultrasound, Hainan Hospital, Chinese PLA General Hospital, Sanya, 572000 China

**Keywords:** Ultrasound guidance, Intracerebral hemorrhage, External drain

## Abstract

We report a new minimally invasive technique utilizing interventional ultrasound for precise external intracerebral hemorrhage drain (EICHD) placement in pigs.

## Background

The severity of cerebral injury is related to the development and formation of a hematoma, which could cause secondary events, including elevated intracranial pressure (ICP) and edema. It is critical that large hematomas are evacuated at an early stage. Catheter drainage-based techniques are notable examples of minimally invasive techniques for intracerebral hemorrhage (ICH) evacuation [1]. These catheter insertion approaches are assisted by many kinds of imaging techniques and can be performed through a small burr hole. However, many procedures cannot be performed at the bedside because they usually require surgical neuronavigation or the assistance of other large imaging equipment. Here, we described a minimally invasive interventional technique using real-time ultrasound guidance through a small burr hole to achieve external intracerebral hemorrhage drainage (EICHD) placement.

## Methods

Three healthy male miniature pigs weighing 10 ± 1 kg were anesthetized by intramuscular injection of 3% pentobarbital sodium (30 mg/kg). We mimicked the clinical conditions of traumatic ICH using an animal model of parenchymal hematoma that was established by stereotactic injection of 3 mL of nonanticoagulated autologous arterial blood into the right lobe of the brain parenchyma 20 mm from the skull [2].

**Fig. 1:**
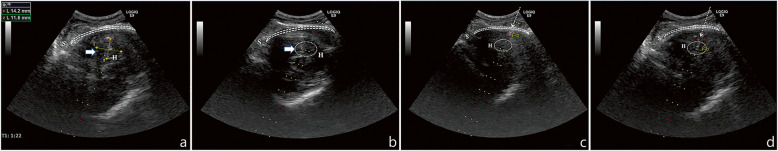
Operative process of ultrasound images showing the target intracerebral hematoma. **a**: The parenchymal hematoma was formed by autologous blood injection (as shown by the white arrow), and the longest diameter is approximately 14.2 mm (as shown by the yellow dotted line 1). **b**: The parenchymal hematoma was identified (as shown by the white arrow and white dotted circle). The ultrasound probe was adjusted so that the center of the puncture guideline passed through the parenchymal hematoma. We know that the distance from the guide channel entrance to the center of hematoma (dot on the image center puncture guideline) is approximately 30 mm. **c**: Real-time tracking of the EICHD following the dotted puncture guideline as indicated by the white dotted arrow. The catheter passed through the burr hole in the skull under real-time ultrasound guidance, and the catheter position is indicated by the yellow arrow. **d**: The catheter reached the correct position in the hematoma. (The catheter is indicated by the white dotted arrow, and the final position is indicated by the yellow arrow). H: Hematoma, S: Skull as shown by the white dotted circle

After the model was established, each brain was subjected to a transcranial ultrasound examination (B-mode and color Doppler) at the back of the ear to identify the parenchymal hematoma (Fig. 1a). After identification of the parenchymal hematoma, the ultrasound probe was adjusted so that the puncture guideline could pass through the center of the parenchymal hematoma, and the exact entry point of the EICHD could also be determined (Additional file 1: Figure S1). The distance from the guide channel entrance to the center of hematoma could be calculated (Fig. 1b). Then, to accommodate the 16-gauge needle (outer diameter 1.6 mm) and catheter (outer diameter 3.0 mm), a 7.0-mm-diameter burr hole was made at the determined entry point using a 7-mm perforator drill under the guidance of the ultrasound. After burr hole establishment, we performed an EICHD insertion under real-time ultrasound guidance (Fig. 1c).

## Results

After the catheter was placed slightly in the parenchymal hematoma (Fig. 1d) and blood could be seen flowing out of the catheter (Additional file 1: Figure S2), the process of EICHD was classified as successful, and the catheter could be positioned and secured well.

## Discussion

Traumatic cerebral injury usually leads to intracerebral hematoma with a mass effect, and EICHD could aid in decompression, which might help to reduce the mortality and disability rate. Recently, with the development of ultrasound equipment, some studies have reported that ultrasound-guided catheter drainage in clinical ICH cases could achieve EVD placement through a burr hole [3]. Therefore, we attempted a new minimally invasive technique of real-time ultrasound-guided EICHD placement with commonly used ultrasonic and other interventional equipment that could be operated at the bedside and resulted in much less invasive damage to the patient.

We have tested a new minimally invasive approach of local hemostatic drug therapy by the real-time guidance of transcranial contrast-enhanced ultrasound (CEUS) based on the same model of traumatic ICH [4]. This study is a continuation and extension of our previous study and forms a set of effective treatment methods for ICH.

However, this new technique also has many limitations. First, because the physiologic window of the skull was limited and the window was relatively narrow, hematomas in all areas of the brain could not be scanned using the current method. Second, this minimally invasive technique could be performed only if the hematoma had been clearly identified. Third, the detection of the hematoma location by transcranial ultrasound was limited due to the two-dimensional images and the limited physiologic window of the skull. Therefore, this technique still requires further investigation.

## Supplementary information

**Additional file 1: Figure S1.** One interventional burr hole is placed for the EICHD catheter, whose positions are adjusted by the ultrasound transducer. **Figure S2.** Blood could be seen flowing out of the catheter.

## Data Availability

Not applicable.

## Abbreviations

EICHD: External intracerebral hemorrhage drain
ICH: Intracerebral hemorrhage
ICP: Intracranial pressure
