# Supplementary material for: Real-time ultrasound-guided external intracerebral hemorrhage drain placement
Source: Mil Med Res. 2020 Jul 2;7:32. doi: 10.1186/s40779-020-00261-9 (PMC7331222; doi:10.1186/s40779-020-00261-9)
Supplement: Supplementary file 1 — Additional file 1: Figure S1. One interventional burr hole is placed for the EICHD catheter, whose positions are adjusted by the ultrasound transducer. Figure S2. Blood could be seen flowing out of the catheter. [file 40779_2020_261_MOESM1_ESM.doc]

Appendix


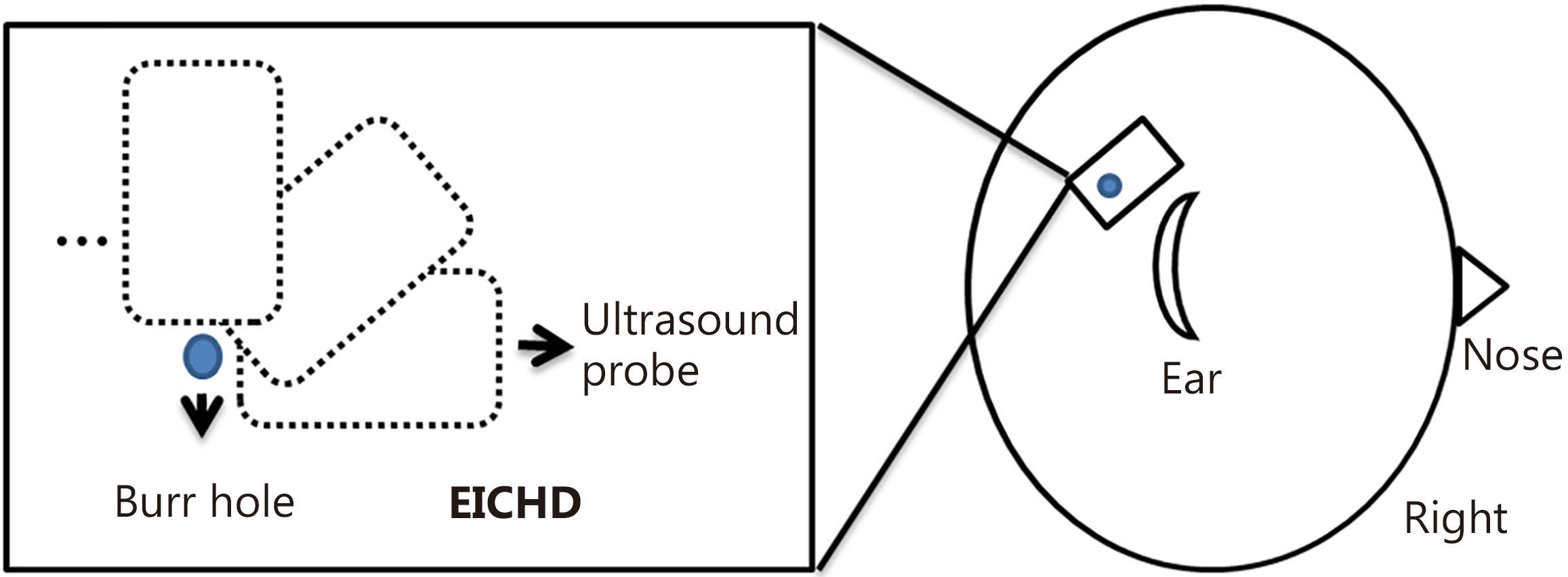


Appendix Figure S1 One interventional burr hole is placed for the EICHD catheter, whose positions are adjusted by the ultrasound transducer. (Right: Right half of the face)


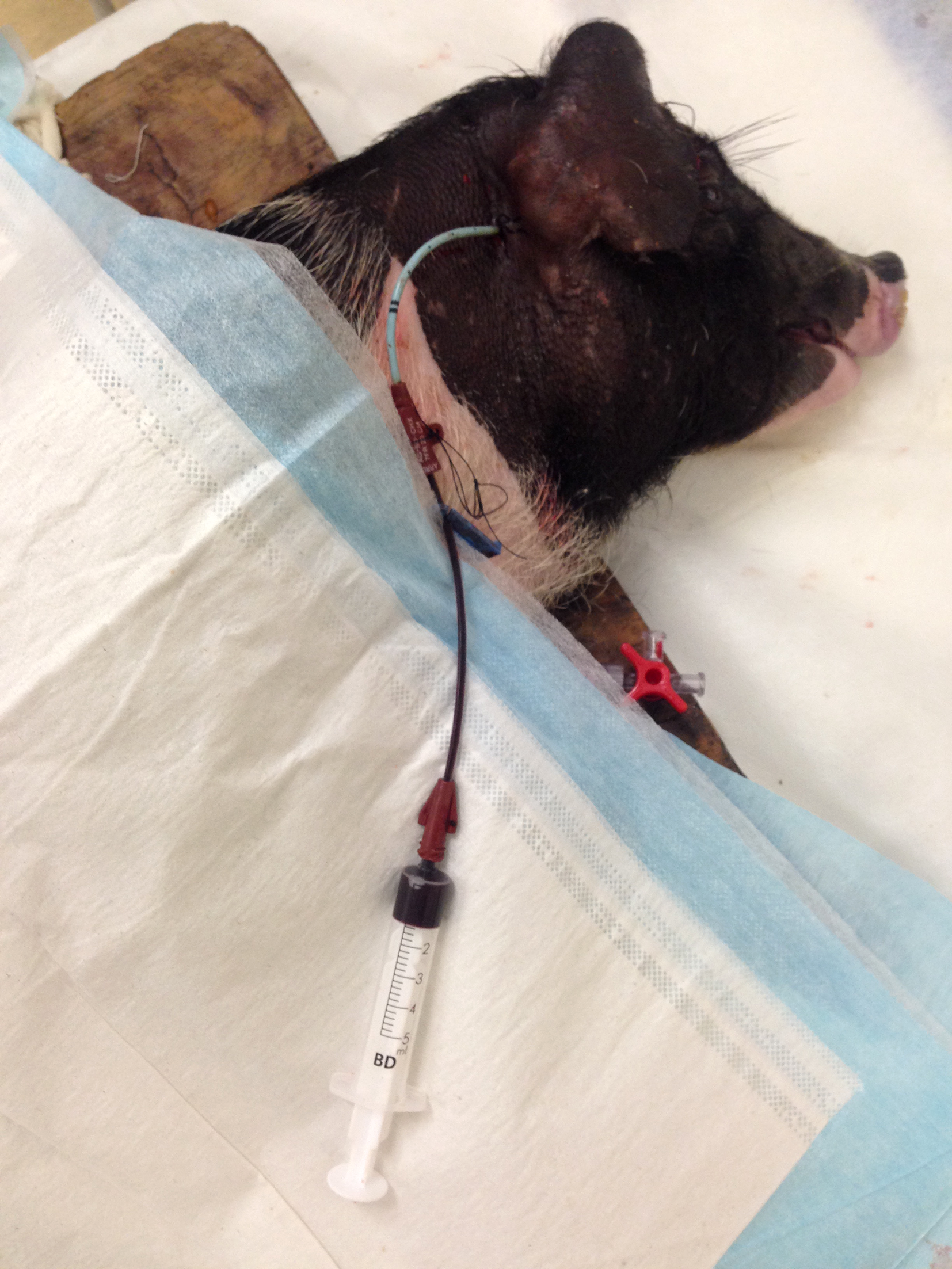


Appendix Figure S2 Blood could be seen flowing out of the catheter.
